# Fast-track surgery applied in gynecological oncological surgical treatment: a prospective randomized trial

**DOI:** 10.4314/ahs.v24i3.32

**Published:** 2024-09

**Authors:** Xunwei Shi, Ling Cui, Yu Shi, Guonan Zhang, Zhirong Yang, Dengfeng Wang

**Affiliations:** Department of Gynaecological Oncology, Sichuan Cancer Hospital & Institute, Sichuan Cancer Center, School of Medicine, University of Electronic Science and Technology of China, Chengdu 610041, Sichuan, China

**Keywords:** fast track surgery, gynaecological surgery, oncological surgery, post-operative length of hospitalization, randomised controlled study

## Abstract

**Background:**

The study aimed to compare the fast-track and traditional gynaecological oncological surgeries from postoperative recovery.

**Methods:**

A total of 107 patients undergoing gynaecological oncological surgery were randomly assigned to the FTS group (n=50) and the traditional group (n=57).

**Results:**

No significant differences in LOS (Length of hospitalization post-operation) were observed. A less total cost of hospitalization and a lower C-Reactive protein (CRP) level were found (P < 0.05). Also, the trial revealed a significantly lower number of the overall complications in the FTS Group (P < 0.05).

**Conclusions:**

FTS applied in gynaecological oncological surgeries is beneficial to patients in terms of the postoperative recovery.

## Introduction

Fast-track surgery (FTS), also known as enhanced recovery after surgery (ERAS), has been widely adopted in various surgical procedures. FTS is a multidisciplinary approach aimed at accelerating recovery and reducing undesirable sequelae from surgical injuries, leading to improved recovery rates and a significant decrease in postoperative morbidity and overall costs 1. Surgical injuries to organs are thought to be mediated by trauma-induced endocrine-metabolic changes and the activation of several biological cascade systems, such as cytokines, complement, arachidonic acid metabolites, nitric oxide, and free oxygen radicals 2. The changes in inflammatory cytokines may reflect the postoperative recovery.

Previously, the use of FTS was not widespread due to concerns about its safety among gynecological oncologists who held conservative views on postoperative feeding times for patients undergoing procedures such as appendectomy or intestinal anastomosis. However, recent studies have shown that FTS is as safe as conventional perioperative care and can accelerate recovery in patients undergoing pancreaticoduodenectomy, leading to a reduction in in-hospital costs. As a result, FTS protocols have been recommended as a solution for rapid recovery after pancreaticoduodenectomy 3. Furthermore, previous studies have demonstrated that FTS is a safe and feasible procedure for liver resection 4. FTS programs have been developed globally with the aim of reducing perioperative stress, improving pain management and gut dysfunction, and minimizing postoperative complications. These efforts can result in hastened patient recovery and reduced time spent in the hospital 5.

As of now, FTS has been successfully implemented globally in various surgical procedures, including gynaecological, colorectal, upper gastrointestinal, and even pancreatic surgeries. However, initial studies on gynaecological surgeries were limited to hysterectomies for benign indications or precancerous lesions 6. Therefore, we conducted a clinical trial to compare the length of hospital stay and post-surgical complications between patients receiving gynaecological oncology surgeries who underwent FTS and those who received traditional management.

## Materials and methods

**Figure 1:**
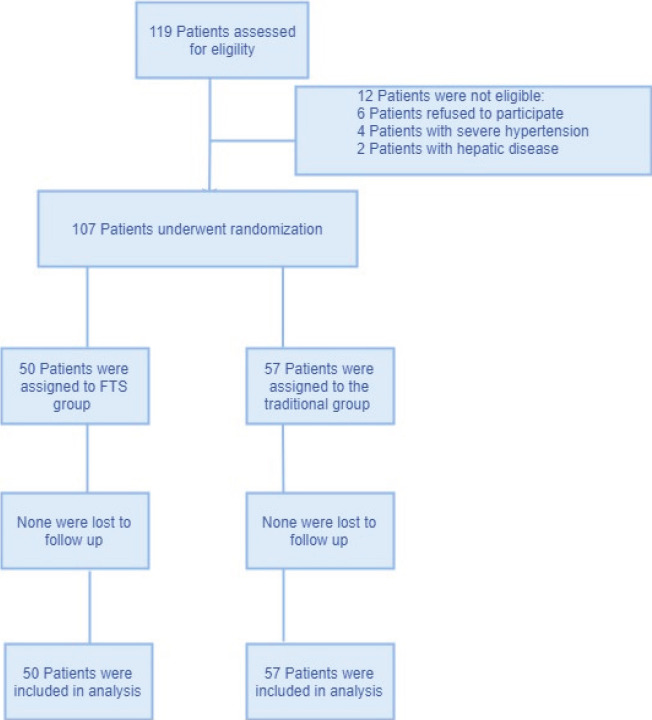
Flow diagram of this study

FTS Database was used to identify patients undergoing gynaecological oncology operations between May 2016 and May 2018 in Sichuan cancer hospital. The FTS protocol, which was already published in previous study 7, was prospectively compared with those of traditional management, as shown in Table 1 (Checklist of fast track and traditional management). 119 Patients were assessed for eligibility and 12 patients were not eligible for severe hypertension, hepatic disease or rejections. And all the eligible patients were randomized to the FTS group and the traditional group by random number table. The surgery was standardised for all the patients, and patients were not blinded to the study. Data collectors were not allowed to participate in the diagnosis and treatment of the patients. The length of postoperative hospitalization (days, mean ± standard deviation), defined as the number of days between the date of discharge and the date of surgery, was selected as the primary endpoint. The secondary endpoints were total cost of hospitalization, and the complications observed during the first three months following the surgery, such as infection, particularly wound infection, lung infection and intraperitoneal infection, as well as postoperative nausea and vomiting, ileus, postoperative haemorrhage and thrombosis. Flow diagram of this study was shown in Figure 1.

**Table 1:** Checklist of fast track and traditional management

	FTS management	Traditional management
Allocation Pre-operative	Computer-generated random numbers	Computer-generated random numbers
	Pre-operative assessment, counselling and FT management education	No FT management education
	Information of the fast-track treatment and the informed consent	Information of the traditional treatment and the informed consent
	Preoperative nutritional drink up to 4 h prior to surgery (Slight liquid diet produce by Methuselah (Shanghai) Medical Technology Co. Ltd)	Pre-operative fasting at least 8h
	Fast solid food before 6 h and liquid food Intake of clear fluids 2 h before anaesthesia	
	Patients are not received mechanical bowel preparation, only oral intestinal cleaner 12 h preoperation can be accepted, but no need of liquid stool	Oral bowel preparation or or mechanical bowl until liquid stool
	Antimicrobial prophylaxis and skin preparation	Antimicrobial prophylaxis and skin preparation
	Preoperative treatment with carbohydrates (10% Glucose 400 ml p.o. 2-3 h before operation) (Patients without diabetes)	No oral intake in the operation day
	
Intraoperative		
	Avoiding hypothermia, keeping the intraoperative core temperature at 36 ±0.5°C	Keeping the intra-operative core temperature at 34.7±0.6°C
	Antiemetics at end of anaesthesia	Not every patient gets antiemetics at end of anaesthesia
Post-operative		
	Postoperative glycaemic control	Postoperative glycaemic control only with diabetes
	Preventive postoperative nausea and vomiting (PONV) control	Postoperative nausea and vomiting (PONV) control when it happens
	Early postoperative diet (3-6 h after surgery, patients resumed a liquid diet, 12 h after surgery patients began to take solid diet)	6 h after surgery, patients resumed a liquid diet, patients began to take solid diet after anal exhaust
	Early mobilisation	Early mobilisation
	Time to drain removal less than 24h (Eliminate postoperative bleeding and urinary fistula, intestinal fistula)	Time to drain removal less than 48h (Eliminate postoperative bleeding and urinary fistula, intestinal fistula)
Audit	Systematic audit improves compliance and clinical outcomes	

### Inclusion criteria

The inclusion criteria for this clinical trial are as follows: Patients who are scheduled to undergo gynaecological oncology surgery, which includes radical hysterectomy and lymphadenectomy, hysterectomy and lymphadenectomy, and cytoreductive procedures for both open and laparoscopic surgery. The patients must be at least 18 years old. Patients must provide signed informed consent.

### Exclusion criteria

The exclusion criteria for this study were as follows: Patients who had a documented infection at the time of surgery were excluded; Patients who were 71 years old or older were excluded due to the high risks associated with surgery, as determined by the ethics committee after thorough discussion; Patients who had ileus at the time of surgery were excluded; Patients with hypocoagulability were excluded; Patients with a history of psychological disorders, alcohol dependence, or drug abuse were excluded; Patients with primary nephrotic or hepatic disease were excluded; Patients with severe hypertension, defined as systolic blood pressure ≥ 160 mmHg and diastolic blood pressure > 90 mmHg, were also excluded.

### Criteria for discontinuing

Criteria for discontinuing the trial include participants who experience unexpected harm or severe adverse events that are likely caused by the trial protocols, or if the ethics committee decides to discontinue their participation due to evidence showing that the risks outweigh the benefits. Participants who are unlikely to complete the trial within a three-month period are also included in the discontinuation criteria.

### Discharge criteria

The following are the criteria for discharge: stable vital signs, alert and oriented state of consciousness, absence of complications or symptoms, ability to walk autonomously, ability to consume solid food, successful passing of flatus, spontaneous urination, good pain control with an NRS < 4 using oral medications, self-sufficiency in performing basic daily activities, and expressed desire by the patient to be discharged.

### Statistical analysis

In the present study, the data, expressed as mean ± SD, were analysed using SPSS 18.0 (IBM Corp., Armonk, NY, USA). LOS, postoperative hemorrhage and thrombosis in the FTS and traditional groups were compared by the student's t-test. The chi-square test or Fisher's exact test was used to analyse the categorical secondary endpoints (complications). P < 0.05 was considered statistically significant.

## Results

As shown in Table 2, a total of 107 patients, 50 for the FTS group and 57 for the control group, were enrolled in this study, with a mean age of 48.62 and 55.25 years, respectively. There were 27 cases of cervical cancer (54%), 14 endometrial cancer (28%), and 9 ovarian cancer (18%) in the FTS group as well as 29 cervical cancer (50%), 9 endometrial cancer (16%) and 19 ovarian cancer (34%) in the traditional group. The subjects from the FTS group and the traditional group measured a similar mean body surface area (1.55 vs. 1.54m2) and the mean body mass index (22.68 vs. 23.17). The operative time (195.30 vs. 200.37 min) and the estimated blood loss during surgery (320.00 vs. 280.18 ml) in the FTS group and the traditional group showed no evident difference as well. Although the mean LOS of the patients from the FTS group was 8.92 days (95% CI: -0.278-1.771), shorter than the traditional group (9.67 days,95% CI: -0.28-1.77). However there was no statistical significance (Table 3). Similarly, according to Table 4, there was no statistical differences between the two groups in the cost of surgical therapy (9703.22 vs. 9538.47 Chinese yuan) and Calcitonin original PCT (0.56vs. 0.52).

**Table 2:** Patient demographic and operative characteristics

	FTS group (50)	Traditional group (57)
Age (years)	48.62	55.25
Disease	50	57
Cervical cancer	27(54%)	29 (50%)
Endometrial cancer	14 (28%)	9 (16%)
Ovarian cancer	9 (18%)	19 (34%)
Body surface area BSA	1.55	1.54
Body Mass Index BMI	22.68	23.17
Operative time (min)	195.30	200.37
Intraoperative blood loss (ml)	320.00	280.18
Laparotomy surgery number	39	48
laparoscopic surgery number	11	9
Blood transfusion number	8	6
Day of drainage	1.44	1.63
Day of fasting	1.02	1.79

**Table 3:** LOS (Length of hospitalization post-operation) between two groups

	FTS group (50)	Traditional group (57)
Mean (Day)	8.92	9.67
Standard deviation	2.03	3.12
95% CI	-0.28-1.77	-0.28-1.77
Significance	0.14	0.15

The total cost of hospitalization was statistically less in the FTS group when compared with the traditional group (38882.44 vs. 42864.12 Chinese yuan; P=0.029). Also, the level of C-Reactive protein (CRP) was 42.13mg/l in the FTS group, significantly lower than 62.50 mg/l in the traditional group (P<0.05) at 2 days after surgery. A similar finding was observed in terms of the days of fasting, which was significantly shorter in the FTS group as compared with the traditional group (1.02 vs1.58; P=0.00), as shown in Table 4.

**Table 4:** Characteristics found to be different between two groups

	FTS group	Traditional group	Significance
Cost of surgical therapy (Chinese *yuan*)	9703.22	9538.47	0.61
PCT (Calcitonin Original, ug/ml)	0.56	0.52	0.84
The total cost (Chinese *yuan*)	38882.44	42864.12	0.03
CRP (C-Reactive protein mg/l) at 2 days after surgery	42.13	62.50	0.00

Postoperative follow-ups revealed that no severe complication was reported in either of the two groups. Table 5 shows that a total of 11 patients developed postoperative complications—three cases in the FTS group (intraperitoneal infection, 2; ileus, 1) and eight cases in the traditional group (wound infection, 3; lung infection, 1; intraperitoneal infection, 4; and ileus,1), including one who had both wound infection and intraperitoneal infection. Also, the number of the complication of infections in the FTS group was less than that in the traditional group (2 vs. 12; sig 0.03).

**Table 5:** Postoperative complications

	FTS group	Traditional group	Significance
Overall Complications	3	13	0.01
Infection	2	12	0.03
wound infection	0	4	2.38
lung infection,	0	1	1.00
intraperitoneal infection,	2	7	0.17
postoperative nausea and	0	0	/
vomiting (PONV)			
Ileus	1	1	1.0
Postoperative haemorrhage	0	0	/
Postoperative thrombosis	0		

## Discussion

In this prospective randomized trial, there was no difference in length of hospital stay (LOS) observed between the two groups. This finding may be attributed to several factors. Compared to general surgical oncology, gynecological oncology surgery typically causes less damage, particularly to the digestive tract, which lowers the likelihood of developing serious complications like digestive tract fistula or gastric content leakage that can be fatal. Moreover, LOS can be influenced by various factors apart from the surgical procedure itself. For example, some patients may require postoperative chemotherapy, which may necessitate longer hospitalization. Additionally, patients who met the discharge criteria during weekends or holidays may not be discharged, making it challenging to accurately analyse the relationship between LOS and the type of surgery performed.

The total cost of hospitalization (Chinese yuan) was significantly lower in the FTS group compared with the traditional group, in spite of the similar cost of surgical therapy which could partly be explained by the operators who were actually the same ones responsible for the surgeries in both groups. Heating blanket employed during the procedure to avoid hypothermia was believed as a major contributor for the lower total cost in the FTS group. Unintentional hypothermia is defined as an accidental low body temperature 8, and the National Institute for Health and Care Excellence (NICE) estimates that 70% of patients admitted to the anesthetic recovery room suffer from hypothermia 9. The accidental perioperative hypothermia is a common event during surgical interventions, usually resulting in an increased rate of perioperative complications such as impaired hemostatic function, delayed wound healing, and some cardiac events 10. In this trial, the intra-operative core-temperature of the patients in the FTS group was maintained stably at 36 ±0.5°C, which significantly shortened the time for anesthesia resuscitation as well as for ventilator application, consequently reduced the costs relevant to these procedures. Meanwhile, the cost for complication treatment was also reduced by fast-track surgery due to the significantly less occurrence of the overall complications and complications of infections. Besides, since early postoperative diet can speed up gastrointestinal motility, days of fasting were much shorter in the FTS group. Patients of ovarian cancer undergoing appendectomy used to fast until anal exhaust according to the protocol of traditional therapy, but early feeding was encouraged in ovarian cancer patients of the FTS group as it has been proved safe and effective in the recently published guidelines aiming at enhanced postoperative recovery after gastrectomy and pancreaticoduodenectomy 11,12. As a result, the early oral feeding and shorter duration of intravenous infusion contributed to the lower total cost in the FTS group as well, owing to the less need for parenteral nutrition, prevention from thrombosis, and some other adjuvant therapies.

The FTS group showed a significantly lower incidence of total postoperative complications and infection rates compared to the traditional management group. This was accompanied by a significant decrease in CRP levels, which is believed to be closely related to good control of insulin resistance, glycemic levels, and intraoperative body temperature. Fasting at midnight can increase insulin resistance, so the FTS patients were given a compound carbohydrate-rich drink designed for use within 2 hours before anesthesia to reduce hunger, thirst, anxiety, and postoperative insulin resistance ^13^. Additionally, perioperative hypothermia prevention is beneficial in reducing stress and wound infections, as demonstrated by numerous meta-analyses and randomized controlled trials on major abdominal surgeries ^14^. The FTS program also shortens preoperative and postoperative fasting time and decreases the amount of bedridden time for patients. This is particularly important for patients with compromised postoperative recovery due to prolonged fasting and confinement to bed, which can lead to negative nitrogen balance ^15^. Early oral feeding, advocated by the FTS protocol, also helps in optimal wound healing. Unrelieved pain can inhibit the immune system, decrease gastrointestinal motility, and cause respiratory dysfunction by increasing oxygen demand ^16^. Therefore, analgesics were given to the FTS group to prevent these pain-induced conditions while allowing mobilization and participation in recovery activities, which are essential parts of the FTS program.

However, the study still had some limitations. This study was conducted on a relatively small sample size of 107 patients, which may limit the generalizability of the findings to the wider population. The present study was conducted in a single center, which may limit the generalizability of the findings to other centers with different patient populations and surgical protocols. Additionally, the study only reported on postoperative complications up to the time of hospital discharge, and longer-term outcomes were not assessed. What's more, the study did not account for potential confounding factors that may have influenced the outcomes, such as preoperative comorbidities or differences in patient characteristics between the two groups. The study did not report on patient-reported outcomes, such as quality of life, satisfaction with care, or postoperative pain, which are important measures of success in surgical interventions. In future studies, it would be beneficial to increase the sample size to improve the generalizability of the findings. Additionally, utilizing a more diverse sample population and controlling for potential confounding variables could strengthen the validity and reliability of the results.

In summary, FTS is a suitable and safe option for patients undergoing gynaecological oncological surgeries that do not harm the digestive tract. This procedure has been shown to be beneficial in improving surgical outcomes by reducing infection-related complications and the overall cost of hospitalization, while also accelerating postoperative recovery.

## Data Availability

The raw data supporting the conclusions of this article will be made available by the authors, without undue reservation.

## List of abbreviations

LOS: Length of hospitalization post-operation
FTS: Fast-track surgery
CRP (FTSC): Reactive protein
